# NIDDK data repository: a central collection of clinical trial data

**DOI:** 10.1186/1472-6947-6-19

**Published:** 2006-04-04

**Authors:** A Jamie Cuticchia, Philip C Cooley, R David Hall, Ying Qin

**Affiliations:** 1 Duke Institute for Genome Sciences and Policy, Duke University, Durham, North Carolina, USA; 2 Bioinformatics Program, RTI International, Research Triangle Park, North Carolina, USA

## Abstract

**Background:**

The National Institute of Diabetes and Digestive and Kidney Diseases have established central repositories for the collection of DNA, biological samples, and clinical data to be catalogued at a single site. Here we present an overview of the site which stores the clinical data and links to biospecimens.

**Description:**

The NIDDK Data repository is a web-enabled resource cataloguing clinical trial data and supporting information from NIDDK supported studies. The Data Repository allows for the co-location of multiple electronic datasets that were created as part of clinical investigations. The Data Repository does not serve the role of a Data Coordinating Center, but rather as a warehouse for the clinical findings once the trials have been completed. Because both biological and genetic samples are collected from many of the studies, a data management system for the cataloguing and retrieval of samples was developed.

**Conclusion:**

The Data Repository provides a unique resource for researchers in the clinical areas supported by NIDDK. In addition to providing a warehouse of data, Data Repository staff work with the users to educate them on the datasets as well as assist them in the acquisition of multiple data sets for cross-study analysis. Unlike the majority of biological databases, the Data Repository acts both as a catalogue for data, biosamples, and genetic materials and as a central processing point for the requests for all biospecimens. Due to regulations on the use of clinical data, the ultimate release of that data is governed under NIDDK data release policies. The Data Repository serves as the conduit for such requests.

## Background

The National Institute of Diabetes and Digestive and Kidney Diseases (NIDDK) of the National Institutes of Health (NIH), part of the U.S. Department of Health and Human Services, have established central repositories for DNA, biospecimens, and data collected in clinical studies. The purpose of the Central Repositories is to increase the utility of NIDDK sponsored research by providing access to the samples and data to a wider research community than those research groups involved in the studies.

The repositories involved in this initiative are:

### Data repository [1]

Research Triangle Institute (RTI International)

Research Triangle Park, NC

### Biosample repository [2]

McKesson Bioservice Corporations (Now Fisher Bioservices)

Rockville, MD

### Genetic (DNA) repository [3]

Rutgers University Cell and DNA Repository

Piscataway, NJ

It is the responsibility of the Data Repository to maintain the linkage of all biological materials to the appropriate clinical data. For ongoing studies, which have not yet released their data, the Data Repository provides the appropriate identifiers to the study coordinators and the specimen repositories in order to create unambiguous linkages between specimens and data.

#### Datasets

As of November 2005, the Data Repository contains data from six completed studies. We also anticipate the availability of an additional five data sets (HEMO, DPP, Virahep-C, EDIC, and CRISP) during 2006. The data sets presently available are:

#### Liver Transplant Database (LTD) [4,5]

Information collected in a sever-year prospective study of 916 liver transplant recipients out of 1563 candidates.

#### Liver Transplant Database Follow-up (LTD2) [4,5]

Long-term follow-up information for the original cohort of liver transplant recipients in the LDT database.

#### National Analgesic Nephropathy Study (NANS) [6]

Information on the relationship between analgesic use and end-stage renal disease.

#### Diabetes Prevention Type 1 Study (DPT-1) [7,8]

Information on the effects of treatment with beta-cell antigens on the onset of Type 1 Diabetes Mellitus.

#### Modification of Diet in Renal Disease (MDRD) [9]

Cooperative study results on the effects of dietary protein and phosphorous and/or reduction of blood pressure and the rate of progression of chronic renal disease.

#### Interstitial Cystitis Data Base (ICDB) [10]

Prospective, longitudinal, multi-center study results of various patient treatments on Interstitial Cystitis.

## Construction and content

### Catalogue

For all data available through the Data Repository, a catalogue entry is maintained. The purpose is to describe to the potential user the data contained in each dataset. It is important that the user have a clear understanding of the datasets because the procedure of requesting data requires the expenditures of time and resources of sources such as an IRB (Institutional Review Board) and examination and approval by both the requestor's organization and NIDDK.

The following information, where available, about each dataset is reported on the web:

### General description

Abstract discussing the study.

### Manuals of operations/protocols

Procedures used in collecting clinical data.

### Forms

Examples of all the forms used in collecting clinical data.

### Data description

Detailed listing of the data available from the study, including SAS variable descriptions.

### Publications

Links to publications resulting from the clinical studies.

**Figure 1:**
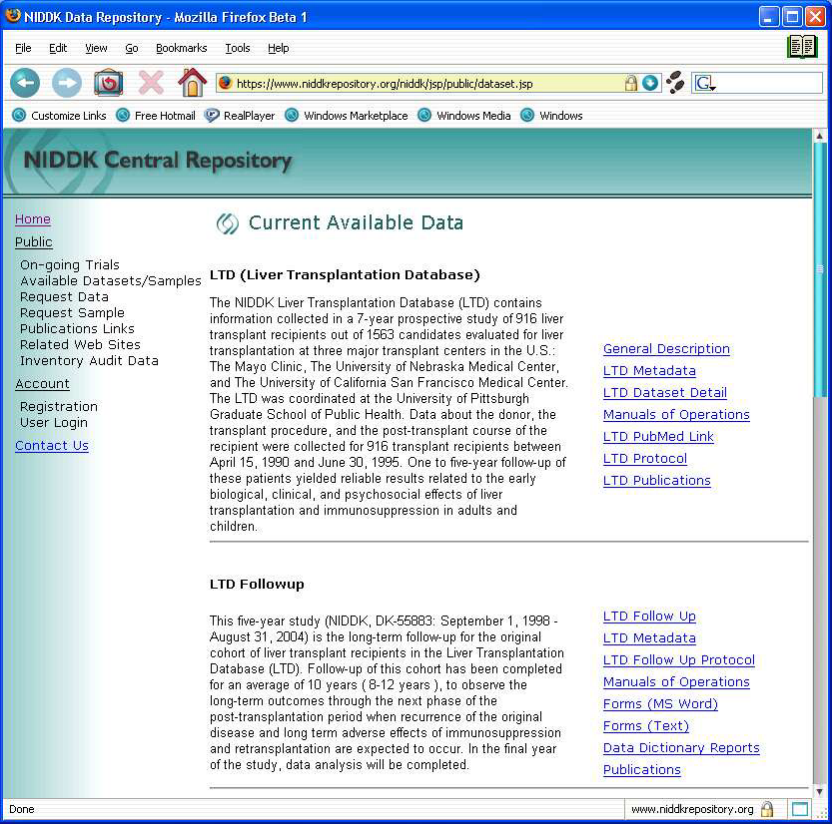
A sample of the listing of currently data available. By selecting the tags to the right of the study abstract, users can obtain more detailed listing on the study.

Figure 1 shows a sample of information available on two datasets.

In addition to the raw data, Data Repository staff work with the studies to develop meta-data descriptions for the data sets. These data include information such as the demographics of the patients, population size, sample availability, and medical tests performed.

## Utility

This information is useful for researchers who may wish to obtain data sets in order to perform meta-studies. One example might be a researcher looking to compare certain clinical data such as creatinine levels over multiple treatments in different studies. An examination of the meta-data would be a first step for the user to determine whether enough information could be obtained from multiple datasets in order to perform a statistically valid analysis.

### Data requests and release

Datasets are provided on CD-ROM. Because of both dataset size and health data security, primary data is not made available through the web. While the datasets are considered to be "limited-use" and exempt from HIPAA, because the data contains patient information (albeit anonymous) it must be treated securely. The NIDDK repository follows the guidelines as specified in the document – *Research Repositories, databases and the HIPAA privacy rule *[11]. Additionally, the Data Repository provides information on available biological specimens.

**Figure 2:**
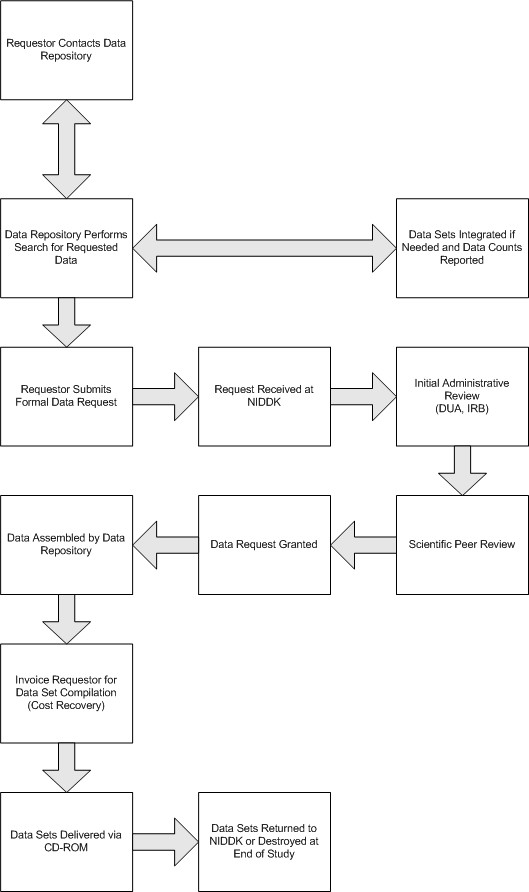
Data Request Process.

Researchers requesting data generally contact the Data Repository to discuss the data sets in more detail. In cases where researchers may wish to obtain data from multiple studies to use for meta-analyses, Data Repository staff work with them to create custom summaries and record counts to determine usefulness. This service is generally provided free or charge, however a nominal fee may be incurred for significant work. Figure 2 show a flow-chart of the data request and release process.

When data are received at the Data Repository, the data undergo an initial review for the assignment of meta-data. The variables in the data sets (generally SAS data) are related to the clinical report forms. During this process a spreadsheet is kept which relate the variables from multiple data sets which are identical measurements. These spreadsheets are used by Data Repository staff as the starting point for answering questions about aggregating data sets. As information is determined through a more vigorous custom analysis, those data are reflected in the spreadsheets. Tools will be constructed as needed for Data Repository staff to automate this process where feasible.

These analyses are performed by internal staff and not data requestors due to the nature of the data privacy. For requestors to perform such analyses would require them to have data use approval for every data set queried in the process. A fee schedule is available at the Data Repository site for these analyses. The work is performed on a cost-recovery basis after an initial analysis.

Once a researcher has determined that the data set(s) meets his/her need, a request for data release is sent to NIDDK for review. NIDDK has developed a panel of reviewers with research experience to conduct these reviews. This review is required for the release of patient data and requires an IRB approval from the requestor's organization and a signed data use agreement specifying the conditions under which the data are to be stored, analyzed and protected. Once approved, the Data Repository prepares the data for release and CD-ROMs are created and mailed. The CD-ROMs, in general, will contain the complete data for all requested datasets and a roadmap that describes the organization of the files on the CD-ROMs. In the case of some meta-studies, only portions of the data may be released. A minimal charge is incurred for the processing and mailing. At the discretion of NIDDK, charges can be waived.

## Discussion

The NIDDK Data Repository discussed here has some unique and innovative features. The effort in NIDDK for the creation of central repositories is still relatively uncommon among NIH funded research. In addition to establishing a mechanism for the retrieval of legacy data and samples from completed studies, the interaction between the central repositories and new studies facilitates future availability of scientific resources.

The information the Data Repository provides is much more than just the clinical results. Value-added work by the Data Repository staff results in the creation of meaningful catalogues of the available data in order to help the researchers determine the utility of the data for their own use. The categorization of data to provide information such as the number of men over 50 which have end-stage renal disease across multiple studies, provide researchers with a head-start in determining which meta-analyses across datasets might be performed.

Another unique feature of the Data Repository is the interaction provided between repository staff and users requesting data. Where predominantly databases provide data with little or no human interaction with the user (*e.g*., web searches), the Data Repository was designed from the start to facilitate interaction of staff and the researchers requesting data. Staff members who are knowledgeable of the studies and the format of the data interact with users at the beginning of the data request process to ensure that the user's needs are met with the available data and that the users have a good understanding of the data formats. Thus, a situation where a user might receive a large number of SAS data files with little more documentation than a list of variable names is precluded. The goal of the Data Repository is to provide as much assistance as necessary for researchers to perform their analyses as efficiently as possible.

In addition to data centralization and user support, the quality control aspect of the Data Repository is a primary feature to this resource. The analysis of data in order to reproduce the findings of the clinical studies (where possible) provides both a check to the accuracy of receipt and completeness of the data. This process also increases the understanding of each dataset at the Data Repository and ultimately results in a more valuable and informed interaction with the users. Users of the Data Repository are asked to provide feedback on the value of the services and this information is relayed to NIDDK.

Finally, the leadership role of the Data Repository in the coordination of a biospecimen collection with clinical data provides an early check in collection process to make certain that data and samples are unambiguously related. In doing so, the Data Repository provides to the clinical studies software tools to track the processing of the samples at the other repositories and their identifiers within the LIMS of the sample repositories for their own use.

### Sample requests

In addition to providing concordance between biosamples and clinical data, the Data Repository acts as the central processing point for DNA and biospecimen requests. Because of limitations in the amount of sample available, requests for biospecimens are rigorously reviewed. Information needed from a researcher requesting samples include a synopsis of the research to be performed, study design, sample management (security) plan, IRB clearance, and data release agreement.

The proposal must state a specific date that an electronic copy of analysis data derived from the received samples is expected to be provided to the NIDDK Central Repositories. The timeframe can be no longer than one year after receipt of samples or upon publication of research in which such data were analyzed, whichever comes first, and annually until the project is completed. The proposal must also state a specific date that the received data are expected to be returned or destroyed. The timeframe can be no longer than 5 years from receipt of the data. Exceptions to the research deadline can be submitted to the repository project officers.

The requestor must submit an IRB clearance or waiver before samples (and associated data) will be released. A copy of the human subjects approval must be attached to the form. An annual human subjects review is required and must be forwarded to maintain eligibility to use the samples.

Since requests require that samples of a particular class of subjects (*e.g*., patients who responded to a particular treatment), the Data Repository staff work with the requestors to determine sample availability. In some cases this may require samples from subjects of different studies, and staff work with requestors to insure that the samples meet their research objectives.

This information is reviewed both by NIDDK staff and a peer-review committee prior to the approval for sample release. The review process also insures that informed consent for the sample release for that particular purpose is in compliance. In general, samples are provided free of charge. The targeted turn-around time for the approval of such requests is two weeks.

## Conclusion

We present here a unique resource for the dissemination of clinical trial data for studies supported by the NIDDK. This resource is growing as new datasets are released and processed for inclusion. The Data Repository provides for the central dissemination, "one-stop-shopping," for clinical data. Moreover, the data validation which occurs during the processing stages helps ensure the accuracy and quality of the datasets. Sample requests are processed by the Data Repository as well.

The Data Repository also provides a service both to ongoing clinical studies and the Biosamples and Genetic (DNA) Repositories. This service includes the assignment of unique site identifiers to ongoing studies and maintaining the linkage between patient data and collected samples.

The success of this resource may set an example to other agencies on the value of providing centralized clinical results and cataloguing available biospecimens during ongoing trials.

## Availability and requirements

The NIDDK Data Repository is freely available for academic and commercial users at . Release of datasets requires approval of NIDDK and the signing of a data use agreement due to the anonymous health information in the records. All comments, suggestions, corrections, and additions, should be sent to NIDDKadmin@rti.org.

## Competing interests

The author(s) declare that they have no competing interests.

## Authors' contributions

AJC coordinated and supervised the project PCC was responsible for the system of site identifiers and liaison to the ongoing studies and suggesting innovative features of the project. RDH was the senior architect in the development of the software. YQ implemented and is the administrator of the NIDDK data repository web site. All authors read and approved the final manuscript.

## Pre-publication history

The pre-publication history for this paper can be accessed here:


